# Comparison of NiO_*x*_ thin film deposited by spin-coating or thermal evaporation for application as a hole transport layer of perovskite solar cells†

**DOI:** 10.1039/d0ra08776a

**Published:** 2020-12-08

**Authors:** Su-Kyung Kim, Hae-Jun Seok, Do-Hyung Kim, Dong-Hyeok Choi, Seung-Ju Nam, Suk-Cheol Kim, Han-Ki Kim

**Affiliations:** School of Advanced Materials Science and Engineering, Sungkyunkwan University 2066, Seobu-ro, Jangan-gu Suwon-si Gyeonggi-do 16419 Republic of Korea hankikim@skku.edu; Korea Electric Power Research Institute Deajeon Republic of Korea ksc5351@kepco.co.kr; School of Mechanical Engineering, Chungnam National University Deajeon Republic of Korea

## Abstract

We compared nickel oxide (NiO_*x*_) deposited by thermal evaporation and that deposited by the spin-coating process, for use in the hole transport layers of inverted planar perovskite solar cells (PSCs). Spin-coating deposition for NiO_*x*_ HTL has been widely used, owing to its simplicity, low cost, and high efficiency. However, the spin-coating process has a technical limit to depositing a large-area uniformly. In contrast, thermal evaporation fabrication has a low price and is able to produce uniform and reproducible thin film. Hence, the chemical states, energy band alignment, surface morphologies, and microstructures of NiO_*x*_ deposited by spin coating and thermal evaporation were analyzed. The PSC with NiO_*x*_ HTL deposited by thermal evaporation showed a higher power conversion efficiency of 16.64% with open circuit voltage 1.07 V, short circuit current density of 20.68 mA cm^−2^, and a fill factor of 75.51% compared to that of PSC with spin-coated NiO_*x*_. We confirmed that thermal evaporation can deposit NiO_*x*_ to give a better performance as a HTL with higher reproducibility than spin-coating.

## Abstract 2

We compared nickel oxide (NiO_*x*_) deposited by thermal evaporation and that deposited by the spin-coating process, for use in the hole transport layers of inverted planar perovskite solar cells (PSCs).

## Graphical abstract

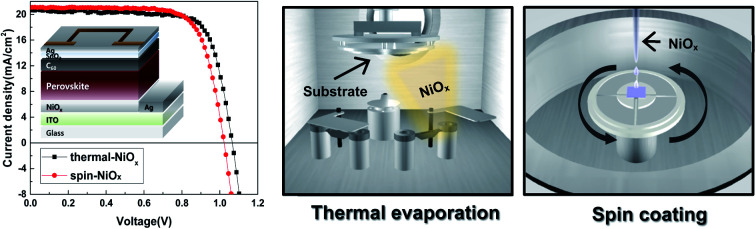

## 1 Introduction

Renewable energy is considered a solution for climate change that is causing critical problems such as a sea level rise, and famine. Among the power generation technologies using renewable energy, the photovoltaic cell is a promising technology that converts infinite solar energy into electrical energy. Organic–inorganic halide perovskite solar cells (PSCs) have received attention owing to a remarkable increase in power conversion efficiency (PCE) from 3.8% in 2009, to 25.2% recently.^1–6^ Organic-inorganic perovskite is represented by the general formula ABX3, where A is an organic/inorganic monovalent cation (methylammonium (CH_3_NH_3_^+^), formamidinium (NH_2_CH_3_NH_2_^+^), Cs^+^ or Rb^+^), B is a divalent metal cation (Pb^2+^ or Sn^2+^), and X is a monovalent halide anion (Cl^−^, Br^−^ or I^−^).^7–9^ Perovskite material is suitable to apply to the solar cell, due to its optical and electronic properties. Perovskite has higher absorption coefficient of *α* > 105 cm^−1^ and lower exciton binding energy of 30–50 meV than other photovoltaic materials. Because of these properties, the light absorption layer of the PSC is able to generate a lot of electric charge, even at relatively thin thickness. Moreover, the charge in the perovskite layer is easy to move to the charge transport layer, because of the high diffusion carrier mobility of 10–60 cm^2^ V^−1^ s^−1^, long carrier lifetime of ∼100 ns and long-range diffusion lengths (∼1 μm) of the perovskite layer.^10–13^ The PSC has a structure by which the active perovskite layer is located between the electron transport layer (ETL) and the hole transport layer (HTL), and PSCs are divided into mesoscopic and planar devices, according to the form of the charge transport layer. PSCs are also divided into n–i–p or p–i–n structures, depending on the direction of incident light. If the incident light is through the HTL, it is defined by p–i–n structure. Among these, the p–i–n planar device has been widely studied, due to advantages such as low cost, easy fabrication process, low temperature process, long-term stability and negligible hysteresis effects.^14–18^

In the interest of improvement in the properties of PSCs, it is important to select the HTL material. From a high-stability point of view, p-type metal oxide with high durability is a suitable candidate for the HTL.^15,19^ One of the most studied p-type metal oxides for HTL is nickel oxide (NiO_*x*_), due to its abundance and chemically stable nature.^16^ Moreover, NiO_*x*_ compounds have large bandgap and a deep-lying valance band that cause favorable energy level alignment with the perovskite active layer.^14,16,20^ NiO_*x*_ can be synthesized by various kinds of methods,^14^ such as solution process,^21,22^ magnetron sputtering process,^23^ pulsed laser deposition,^24^ and electron-beam-evaporation.^25^ Among them, spin-coating, using NiO nanoparticles suspension, has been widely used, owing to its simplicity, and low cost. For example, Liu *et al.* fabricated NiO_*x*_ layers using spin-coating for the HTL of PSCs, and obtained a PCE of 15.89%.^26^ However, the spin-coating process has a technical limit to uniform deposition large-area and upscaling for solution-based processes is difficult to optimize, because it is greatly affected by deposition condition, such as wetting behavior and solvent evaporation.^27^ Although magnetron sputtering, atomic layer deposition, and laser deposition can obtain uniform and reproducible NiO_*x*_ thin films, the processing cost is highly increased.^28^ But the thermal evaporation is a low-cost fabrication process, and it is able to obtain the desired uniformity and reproducibility of thin film.^29^

In this work, we compared properties, such as structure, energy band level, transmittance, and surface morphology, of NiO_*x*_ film deposited by thermal evaporation or spin-coating processes on ITO electrode. NiO_*x*_ deposited by thermal evaporation (thermal-NiO_*x*_) and NiO_*x*_ deposited by spin-coating (spin-NiO_*x*_) were applied to PSCs as HTL, and their photovoltaic performances were compared to substitute typical spin-NiO_*x*_ HTL with thermal-NiO_*x*_.

## 2 Experimental

### 2.1 Preparation of NiO_*x*_ thin films

**Fig. 1:**
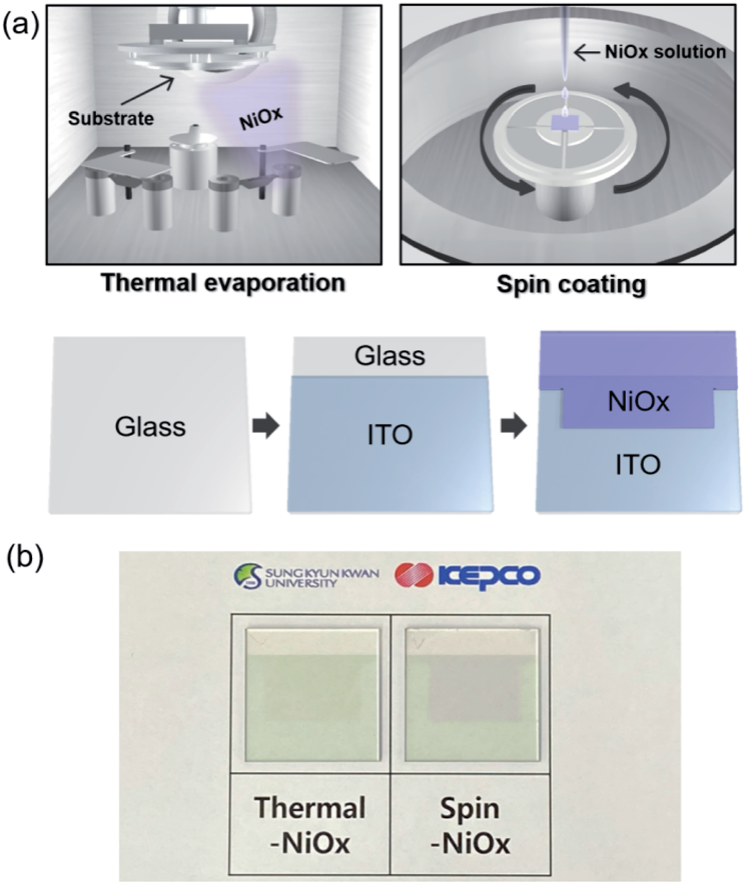
(a) Schematic of the fabrication process of the patterned NiO_*x*_/ITO anode on glass for the perovskite solar cell. (b) Photograph of NiO_*x*_/ITO samples deposited by thermal evaporation or spin-coating.

Fig. 1(a) shows a schematic fabrication process by which NiO_*x*_ is deposited by thermal evaporation and spin-coating processes on the indium tin oxide (ITO) anode. The substrate (2.5 × 2.5 cm^2^) with pre-coated ITO anode underwent UV/ozone treatment for 30 min before the deposition of thermal-NiO_*x*_, and coating of spin-NiO_*x*_ HTL on top of ITO anode. Thermal-NiO_*x*_ was deposited 5 nm thick by evaporating NiO granules (>99.9%, 3–12 mm) in the thermal evaporator maintaining a vacuum condition of 1 × 10^−6^ torr and deposition rate less than 0.2 Å s^−1^. We conducted study on the performance of perovskite solar cell according to thickness of thermal-NiO_*x*_, as shown in Fig. S1,† and the optimum thickness was set at 5 nm based on the results. Spin-NiO_*x*_ was deposited that was 30 nm thick by spin-coating NiO nanoparticle solution at 4000 rpm for 40 s, then, it was annealed at 300 °C for 30 min in air. Fig. 1(b) shows a photograph of each sample after deposition.

### 2.2 Characterization of the NiO_*x*_ HTL

The chemical states of NiO_*x*_ thin films were investigated by a X-ray photoelectron spectroscopy (K-alpha, Thermoscientific). The energy band alignments of NiO_*x*_ thin films were confirmed by UV/visible spectroscopy (Lambda 750, PerkinElmer) and ultraviolet photoelectron spectroscopy (NEXSA, Thermoscientific) measurement. The surface morphologies of NiO_*x*_ thin films and perovskite layers were investigated by field emission scanning electron microscopy (JSM-7600F, JEOL) and atomic force microscope (n-Tracer, NanoFocus) analysis. The microstructures and interface between NiO_*x*_ thin film and perovskite were analysed by high resolution transmittance electron microscope (JEM-2100F, JEOL). The device performance of solar cells with NiO_*x*_ HTL was measured using a solar simulator (Oriel Sol3A, Newport) with AM 1.5 G irradiation.

### 2.3 Fabrication and evaluation of PSCs with NiO_*x*_ HTL

To evaluate the performance of NiO_*x*_ as HTL of PSCs, p–i–n PSCs was fabricated. Immediately before perovskite deposition, thermal-NiO_*x*_ was annealed continuously annealed from room temperature to 330 °C and annealed for 1 min after reaching 330 °C. Because, as shown Fig. S2,† thermal-NiO_*x*_ has poor wettability for perovskite solution. Then, the perovskite layer was deposited on NiO_*x*_ HTL from a precursor solution prepared by mixing formamidinium iodide (FAI, 1.09 M), cesium iodide (CsI, 0.25 M), methylammonium bromide (MABr, 0.11 M), lead(ii) iodide (PbI_2_, 1.23 M), and lead(ii) bromide (PbBr_2_, 0.22 M) in *N*,*N*-dimethylformamide (DMF) and dimethylsulfoxide (DMSO) (8 : 2 v/v). The perovskite precursor solution was spin-coated by continuous two-step process at 500 rpm for 5 s, and 5000 rpm for 45 s. The perovskite precursor solution was spin-coated by continuous two-step process at 500 rpm for 5 s, and 5000 rpm for 45 s. During the second coating step, 0.4 mL of anhydrous chlorobenzene was dropped onto the spinning substrate after 15 s, then annealed at 100 for 30 min. After that, C_60_, SnO_2_, and Ag layers were sequentially deposited on a perovskite layer by thermal evaporation process. As electron transport layer, C_60_ was deposited 35 nm with deposition rate of 0.6 Å s^−1^ and SnO_2_ was deposited 3.5 nm with rate 0.1 Å s^−1^. Finally, 120 nm Ag was deposited as electrode on top. All thermal evaporation processes were performed in maintaining a vacuum condition of 1 × 10^−6^ torr.

## 3 Results and discussion

### 3.1 Chemical states of NiO_*x*_ thin films

**Fig. 2:**
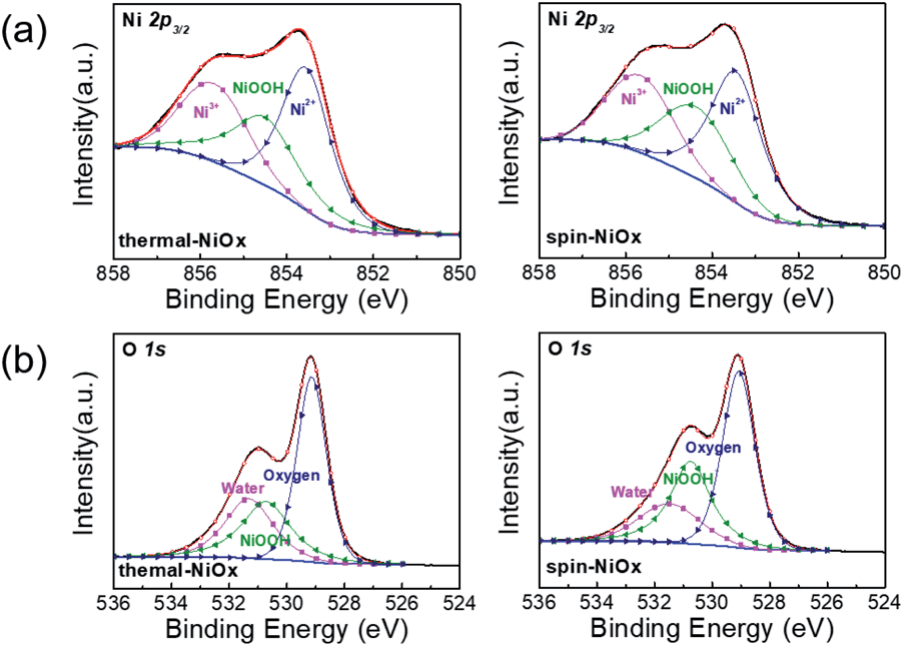
XPS spectrum of (a) Ni 2p peaks and (b) O 1s peaks of the NiO_*x*_ thin film by thermal evaporation and spin coating.

The X-ray photoelectron spectroscopy (XPS) measurements were performed to compare the chemical states and stoichiometries of thermal-NiO_*x*_ and spin-NiO_*x*_ HTL. Because of its wide bandgap, the stoichiometric form of NiO is an insulator with a very low intrinsic conductivity of 10^−13^ cm^−1^ at room temperature.^30,31^ But, NiO is a metal-deficient p-type semiconductor, so Ni vacancies are present at the cation lattice site. Due to Ni vacancies, some Ni^2+^ ions have to be converted to Ni^3+^ ions in order to maintain the electrical neutrality in the structure, and the created Ni^3+^ ions take charge of conduction in NiO.^32–36^ The creation of Ni^3+^ from Ni^2+^ can be expressed according to the following reaction:^36^1
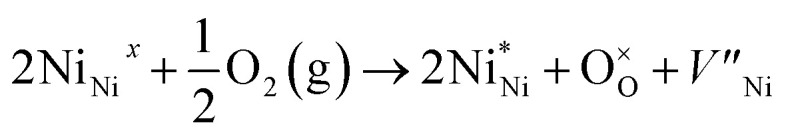
where, two Ni^2+^ ions (Ni_Ni_^*x*^) react with oxygen gas, then create two Ni^3+^ ions 
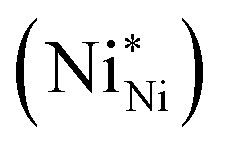
 and ionized nickel vacancy 
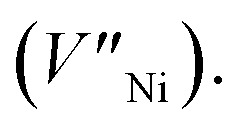
 The created nickel vacancy and Ni^3+^ ions in the NiO crystal serve as hole acceptors.^36^Fig. 2 shows the XPS spectra of Ni 2p and O 1s. In the Ni 2p_3/2_ XPS spectra of both thermal-NiO_*x*_ and spin-NiO_*x*_, three peaks are observed, which are located at binding energies of 853.5 eV, 854.5 eV and 855.8 eV. These peaks correspond to the Ni^2+^, nickel-oxyhydroxide (NiOOH), and Ni^3+^, respectively. The reason for the NiOOH peak being observed at 854.5 eV, is that the surfaces of both of the samples were hydroxylated, due to atmospheric exposure. The integrated peak area ratio of Ni^3+^/Ni^2+^ in the NiO_*x*_ films calculated from XPS data was equal to 0.69 for both the thermal-NiO_*x*_ and spin-NiO_*x*_. In the oxygen O 1s spectra, the peaks located at 529.1 eV, 530.8 eV and 531.5 eV correspond to oxygen, NiOOH, and water, respectively. The peak area corresponding to the NiOOH of spin-NiO_*x*_ is larger than that of thermal-NiO_*x*_, because the solution process is exposed to more moisture and air than the vacuum process. This result is consistent with the tendency that spin-NiO_*x*_ has better wettability for perovskite solution, shown in Fig. S2.† But when NiOOH is formed on the surface of a NiO_*x*_ thin film, the stability and conductivity may be decreased: therefore, it is necessary to control NiOOH on the surface of NiO_*x*_ thin film.^37,38^ XPS analysis results indicate that stability and conduction properties of thermal-NiO_*x*_ are better than spin-NiO_*x*_, because the intergrated peak area ratios of Ni^3+^/Ni^2+^ of both samples are the same, but spin-NiO_*x*_ contain more amount of NiOOH.

### 3.2 Energy band alignment of NiO_*x*_ thin films for HTL

**Fig. 3:**
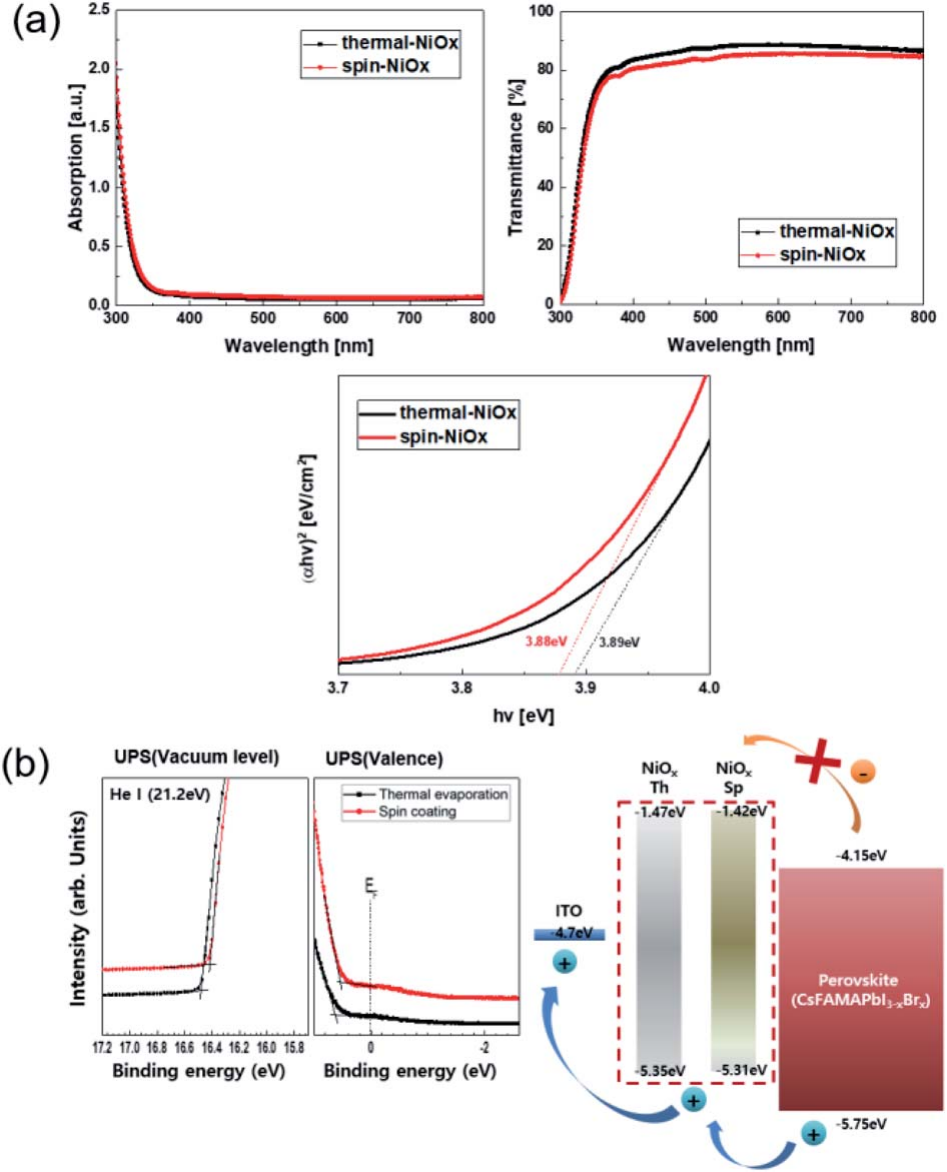
(a) Absorbance and transmittance of thermal-NiO_*x*_ and spin-NiO_*x*_ thin film deposited on the glass. And optical energy bandgap of thermal-NiO_*x*_ and spin-NiO_*x*_. (b) UPS (He I) spectra and schematic illustration of energy band levels of NiO_*x*_ with different coating processes.

Fig. 3(a) shows the absorbance and transmittance of NiO_*x*_ thin films that were measured by UV/Vis spectroscopy. The transmittance of thermal-NiO_*x*_ was slightly higher than that of spin-NiO_*x*_, because the thickness of thermal-NiO_*x*_ is thinner than that of spin-NiO_*x*_. The optical energy band gap is calculated using the equation shown below:

2 *αhν* = *A*(*hν* − *E*_gap_)^1/2^

where *α*, *hν*, *E*_gap_ and *A* are the absorption coefficient, incident photon energy, optical energy band gap, and constant, respectively.

The calculated energy band gap values of thermal-NiO_*x*_ and spin-NiO_*x*_ are 3.88 eV and 3.89 eV respectively, and the values are very similar. The ultraviolet photoelectron spectroscopy (UPS) measurement was conducted to analyze the properties of the energy band of NiO_*x*_ as shown Fig. 3(b). Although there was no noticeable difference in the highest occupied molecular orbital (HOMO), thermal-NiO_*x*_ showed a slightly lower HOMO than spin-NiO_*x*_. Since *V*_oc_ increases when HTL with deeper HOMO levels are applied to PSCs,^10,39–41^ it is anticipated that the perovskite device with thermal-NiO_*x*_ applied has a slightly higher *V*_oc_.

### 3.3 Surface morphologies and microstructures

**Fig. 4:**
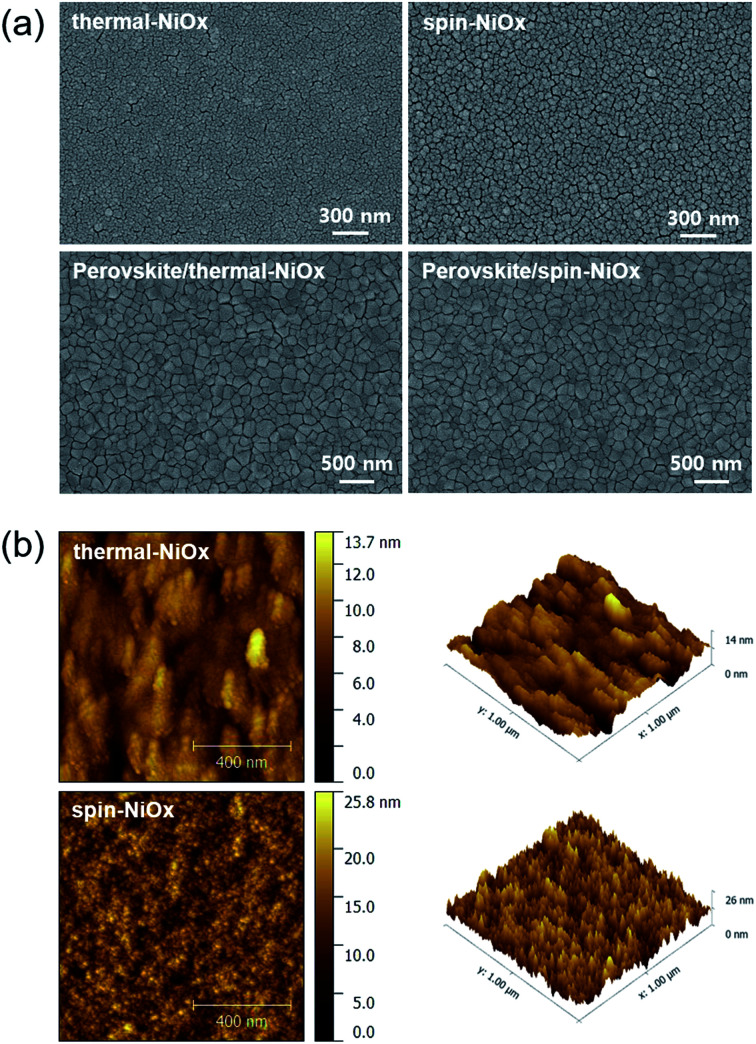
Comparision between thermal-NiO_*x*_ and spin-NiO_*x*_ morphologies: (a) SEM images of NiO_*x*_ and perovskite deposited on NiO_*x*_, (b) surface image measured by AFM of NiO_*x*_ deposited using different deposition processes.

The surface morphologies of NiO_*x*_ thin film and perovskite layer were investigated by scanning electron microscopy (SEM) and atomic force microscope (AFM). Fig. 4(a) shows that compared to the surface morphology of spin-NiO_*x*_, thermal-NiO_*x*_ is denser, and has smaller grain size. The grain size of thermal-NiO_*x*_ is about 25 nm, but the spin-NiO_*x*_ is approximately 50 nm, twice the size of thermal-NiO_*x*_. However, the perovskite layer, coated on the NiO_*x*_ thin films deposited by different processes, shows a similar surface morphology regardless of the NiO_*x*_ deposition process, and the grain size is about 200–250 nm. Fig. 4(b) shows the surface morphology image measured by AFM. Spin-NiO_*x*_ has a rougher surface than thermal-NiO_*x*_. The root mean square (RMS) and actual surface area value were calculated through the AFM results. The RMS value of spin-NiO_*x*_ is 3.29 nm, which is about twice that of the thermal-NiO_*x*_ (RMS 1.74 nm), and spin-NiO_*x*_ has a large actual surface area in the same project area. The actual surface area of the spin-NiO_*x*_ was 1.12 μm^2^ and that of the thermal-NiO_*x*_ was 1.00 μm^2^ per project area (1.00 μm^2^). Therefore, it is estimated that when NiO_*x*_ is deposited by spin-coating process using nanoparticles, it has a larger contact area with the perovskite layer. The large contact area between the perovskite layer and HTL reduces charge recombination, and contributes to efficient charge carrier transport.^42,43^ In contrast, NiOOH present in a large surface area may adversely affect hole transport.

**Fig. 5:**
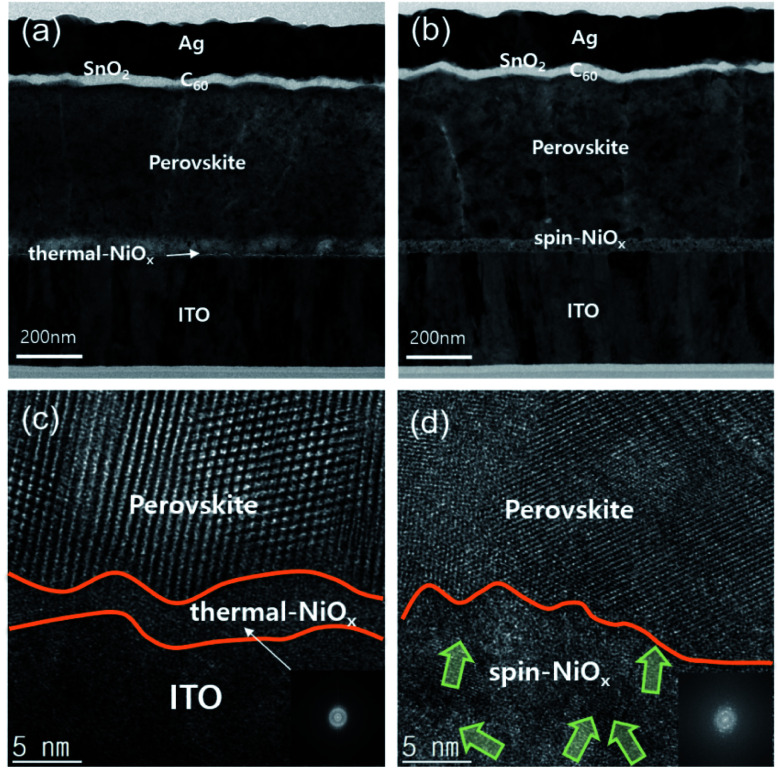
Cross-sectional TEM image obtained from perovskite solar cell with (a) the thermal-NiO_*x*_ HTL, (b) the spin-NiO_*x*_ HTL. And enlarged HR-TEM images obtained from (c) interface between thermal-NiO_*x*_ and the perovskite active layer, and interface between ITO and thermal-NiO_*x*_, (d) interface between spin-NiO_*x*_ and perovskite active layer.

To compare the microstructures and interface of thermal-NiO_*x*_ and spin-NiO_*x*_, high resolution transmission electron microscopy (HR-TEM) was performed and Fig. 5 shows the measured image. Fig. 5(a) and (b) are cross-sectional TEM images of the inverted-planar PSCs with thermal-NiO_*x*_ or spin-NiO_*x*_ HTL. All interlayer interfaces of PSCs were well distinguished, and no diffusion at the interface was observed. Fig. 5(c) is enlarged HR-TEM images from the interface between the thermal-NiO_*x*_ and the perovskite layer. Fig. 5(c) reveals that the crystallization of NiO_*x*_ has not occurred, since the thickness of thermal-NiO_*x*_ is very thin (about 5 nm). The diffuse fast Fourier transform (FFT) pattern also confirms the amorphous structure of thermal-NiO_*x*_. On the other hand, Fig. 5(d) shows that spin-NiO_*x*_ consists of nanoparticles grown in random directions and there is a well-defined interface between NiO_*x*_ and the perovskite layer, without diffusion at the interface. Meanwhile, thermal-NiO_*x*_ has a smoother interface with perovskite than the spin-NiO_*x*_ which is consistent with the AFM results.

### 3.4 Performance of the PSCs contained NiO_*x*_ HTL

**Fig. 6:**
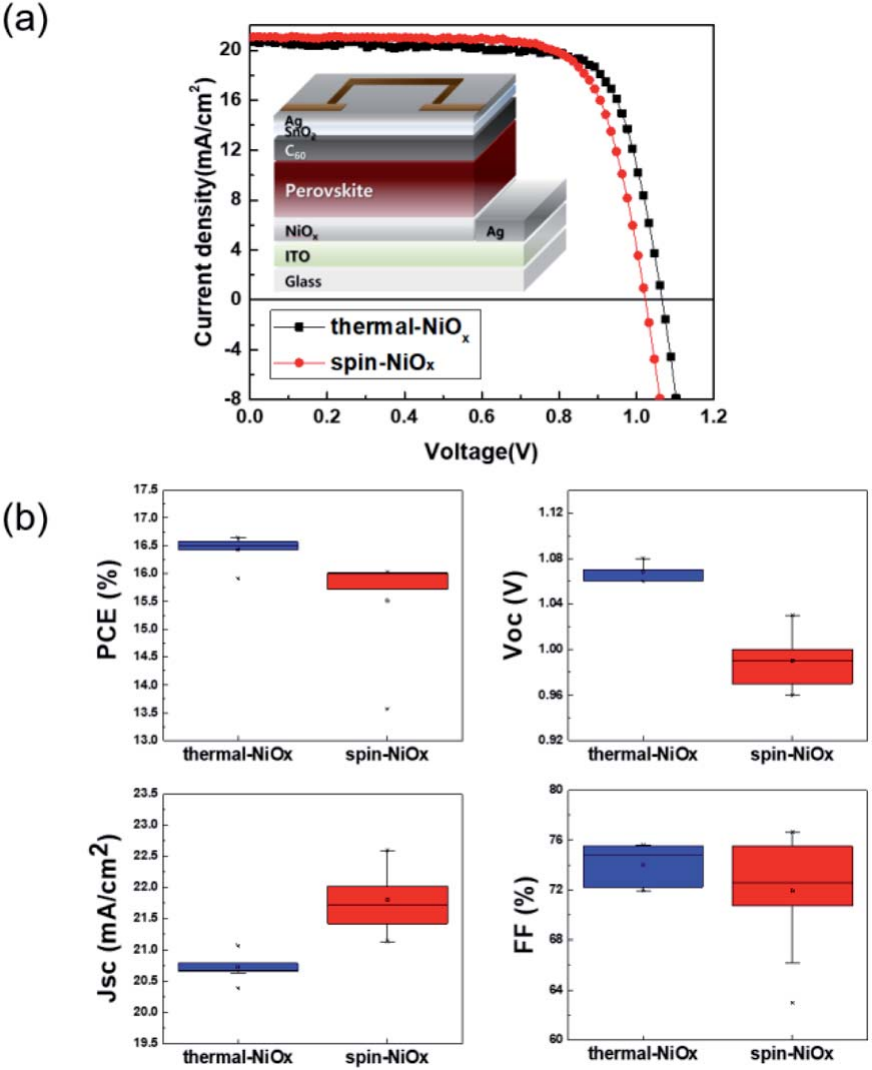
(a) Current density–voltage curves and schematics of perovskite solar cell with NiO_*x*_ for hole transport layer, (b) box chart representation of photovoltaic parameters of perovskite solar cells.

Fig. 6(a) shows the structure of the device that was fabricated to confirm the photoelectric conversion characteristics of the PSC that was applied with NiO_*x*_ as HTL. And Fig. 6(a) shows the *J*–*V* curves of the best performing PSC with each of the thermal-NiO_*x*_ and spin-NiO_*x*_. The best power conversion efficiency (PCE) of PSCs with thermal-NiO_*x*_ was 16.64% (with *V*_oc_ = 1.07 V, *J*_sc_ = 20.68 mA cm^−2^ and FF = 75.51%), and that with spin-NiO_*x*_ was 16.02% (with *V*_oc_ = 1.02 V, *J*_sc_ = 21.14 mA cm^−2^ and FF = 74.14%). Forward and reverse *J*–*V* curves of thermal-NiO_*x*_ and spin-NiO_*x*_ were shown in Fig. S3.† PSCs with thermal-NiO_*x*_ showed little hysteresis, but that with spin-NiO_*x*_ showed changes in *V*_oc_ and FF. It is estimated that because spin-NiO_*x*_ is less stable due to contain more NiOOH than thermal-NiO_*x*_, as shown in the XPS data. Fig. 6(b) is a box plot showing the distribution of each parameter obtained from the measurement of numerous *J*–*V* curves of the PSCs. The average photovoltaic parameter value of PSCs with thermal-NiO_*x*_ was PCE = 16.43%, *V*_oc_ = 1.07 V, *J*_sc_ = 20.73 mA cm^−2^ and FF = 74.00%, whereas that with spin-NiO_*x*_ was PCE = 15.51%, *V*_oc_ = 0.99 V, *J*_sc_ = 21.80 mA cm^−2^ and FF = 15.51%. The *V*_oc_ of PSC using thermal-NiO_*x*_ was higher than that with spin-NiO_*x*_, which is presumed to be because the HOMO level of thermal-NiO_*x*_ has a deeper value than that of spin-NiO_*x*_, as shown in Fig. 3(b). In contrast, *J*_sc_ of PSCs using spin-NiO_*x*_ was higher than that using thermal-NiO_*x*_. It is assumed to be because the perovskite layer of PSC with spin-NiO_*x*_ is exposed to large amount of light than that of PSC with thermal NiO_*x*_, as shown in Fig. S4.† Fig. S4† shows the optical transmittance spectra of spin-NiO_*x*_/ITO/glass, thermal-NiO_*x*_/ITO/glass, and ITO/glass, and the transmittance values are 75.7%, 74.2%, and 77.4% respectively in 300 nm–800 nm. The PCE was higher for PSCs including thermal-NiO_*x*_, and all parameters of spin-NiO_*x*_ have a wider distribution than those of thermal-NiO_*x*_. The results show that the thermal evaporation process is advantageous for obtaining a thin film with higher reproducibility than the spin-coating process.

## 4 Conclusions

In summary, we compared a thermal evaporated NiO_*x*_ and spin-coated NiO_*x*_ to apply the hole transport layers of PSCs. We evaluated the chemical states, energy band alignments, surface morphologies, and microstructure properties of thermal-NiO_*x*_ and spin-NiO_*x*_ thin film. While the chemical states and energy band gaps of thermal-NiO_*x*_ and spin-NiO_*x*_ were not significantly different, thermal-NiO_*x*_ had a slightly deeper HOMO level than did spin-NiO_*x*_. But the surface morphologies are different between thermal-NiO_*x*_ and spin-NiO_*x*_ with the grain size and RMS of spin-NiO_*x*_ being about twice as large as that of thermal-NiO_*x*_, and the contact area with perovskite layer also being larger. To evaluate the characteristics for HTL, PSCs were fabricated with thermal-NiO_*x*_ or spin-NiO_*x*_ and the photovoltaics parameters measured. The PCE of PSCs with thermal-NiO_*x*_ was higher than that with spin-NiO_*x*_ and the reproducibility was better. Consequently, we confirmed the performance as HTL of NiO_*x*_ that was deposited by the thermal evaporation process, and confirmed that the thermal evaporation process is more reproducible than the solution process. Therefore, the thermal evaporation processed NiO_*x*_ HTL enables large-scale, economic, and reliable fabrication for next generation.

## Conflicts of interest

There are no conflicts to declare.

## Supplementary Material

RA-010-D0RA08776A-s001
