# Supplementary material for: Comparison of NiOx thin film deposited by spin-coating or thermal evaporation for application as a hole transport layer of perovskite solar cells
Source: RSC Adv. 2020 Dec 8;10(71):43847–52. doi: 10.1039/d0ra08776a (PMC9058328; doi:10.1039/d0ra08776a)
Supplement: RA-010-D0RA08776A-s001 [file RA-010-D0RA08776A-s001.pdf]

Electronic Supplementary Material (ESI) for RSC Advances.
This journal is © The Royal Society of Chemistry 2020

## Supporting Information

# Comparison of $NiO_x$ thin film deposited by spin-coating or thermal evaporationfor applicationas a hole transport layer of perovskite solar cells

Su-Kyung Kim[a,b], Hae-Jun Seok[a], Do-Hyeong Kim[b], Dong-Hyeok Choi[a,b] ,Seung-Ju Nam[c], Suk-Cheol Kim*[b], and Han-Ki Kim*[a]

[a]School of Advanced Materials Science and Engineering, Sungkyunkwan University, 2066, Seobu-ro, Jangan-gu, Suwon-si, Gyeonggi-do 16419, Republic of Korea.
[b]Korea Electric Power Researc Institute, Deajeon, Republic of Korea.
[c]School of Mechanical Engineering, Chungnam National University, Deajeon, Republic of Korea.

[a] E-mail: hankikim@skku.edu Fax: +82-31-201-2462; Tel: +82-31-205-2462
[b] E-mail: ksc5351@kepco.co.kr Fax: +82-61-345-3047; Tel: +82-61-345-3010

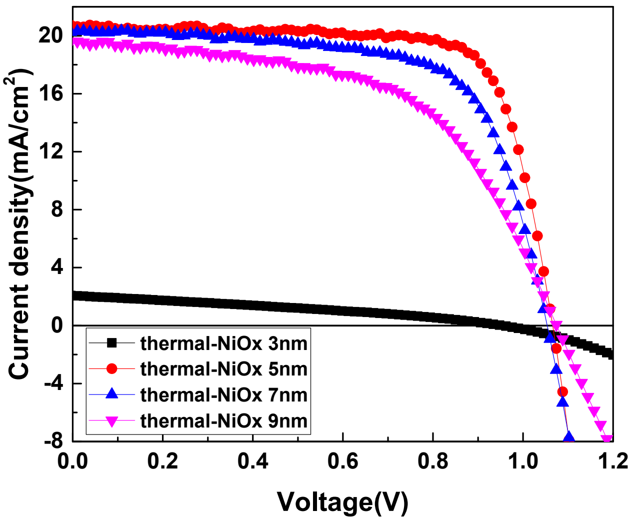


**Fig. S1** J-V curves of perovskite solar cells with thermal-$NiO_x$ according to the different thickness of thermal $NiO_x$.

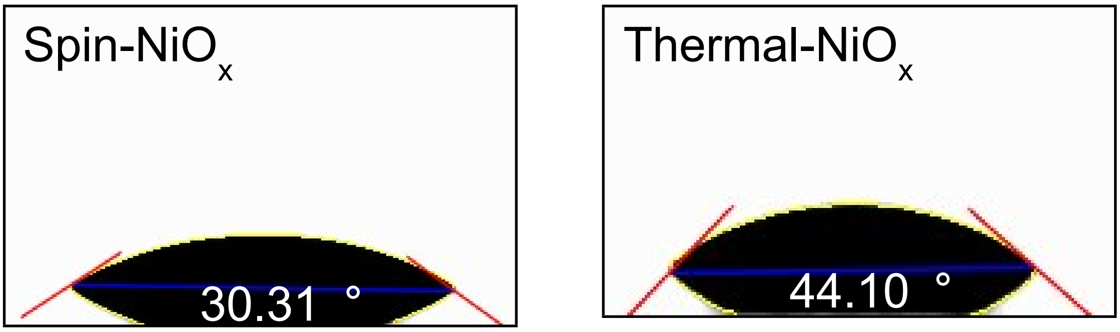


**Fig. S2** Contact angles of (a) the spin-$NiO_x$ and (b) the thermal-$NiO_x$ for perovskite precursor solution.

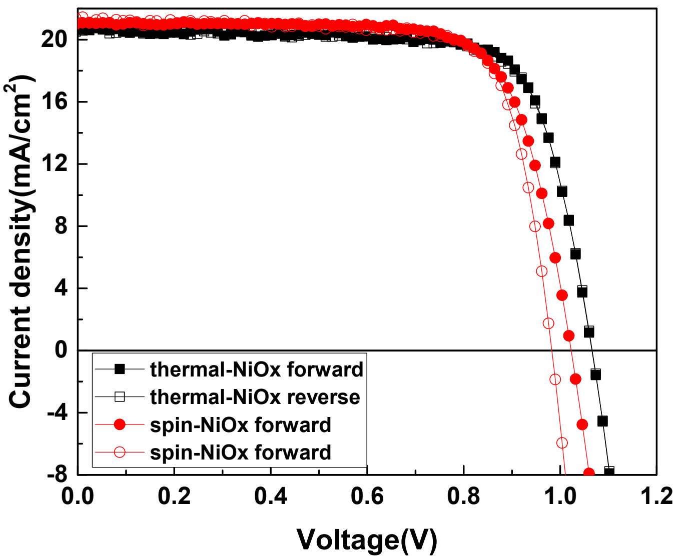


**Fig. S3** Forward and reverse J-V curves of the best performing PSCs with each of thermal-$NiO_x$ and spin-$NiO_x$.

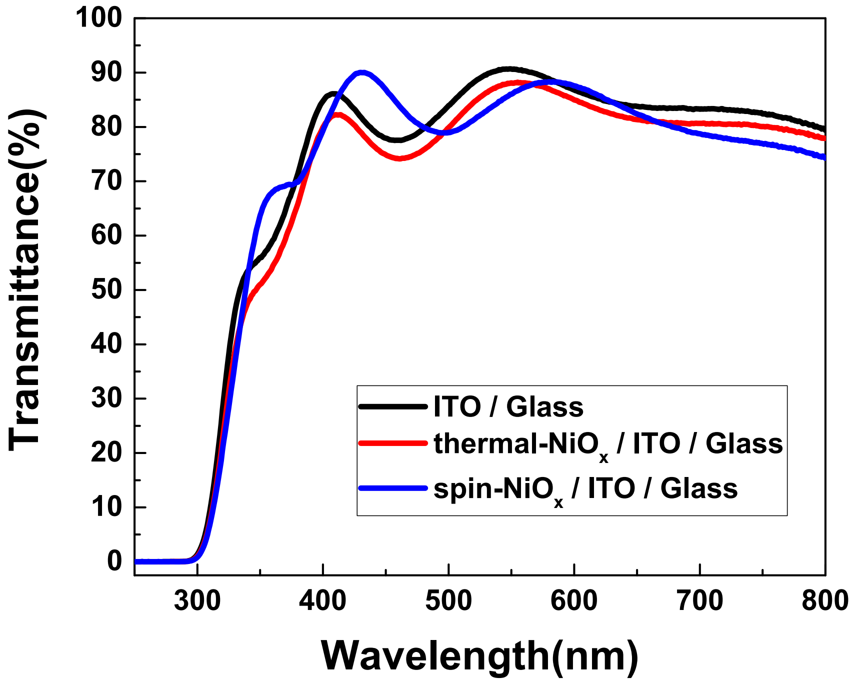


**Fig. S4** Optical transmission spectra of spin-$NiO_x$ or thermal-$NiO_x$ on ITO/Glass and ITO/Glass at 200~800nm wavelength.
